# Serum Uric Acid Levels and Cerebral Microbleeds in Patients with Acute Ischemic Stroke

**DOI:** 10.1371/journal.pone.0055210

**Published:** 2013-01-25

**Authors:** Wi-Sun Ryu, Chi Kyung Kim, Beom Joon Kim, Seung-Hoon Lee

**Affiliations:** 1 Department of Neurology, Seoul National University Hospital, Seoul, Republic of Korea; 2 Clinical Research Center for Stroke, Clinical Research Institute, Seoul National University Hospital, Seoul, Republic of Korea; 3 Department of Neurology, Seoul National University Bundang Hospital, Gyeonggi-do, Republic of Korea; Kaohsiung Chang Gung Memorial Hospital, Taiwan

## Abstract

Unlike experimental studies indicating a neuroprotective property of uric acid, clinical studies have shown that elevated levels of uric acid are associated with a risk of ischemic stroke. However, the association of uric acid with cerebral hemorrhage has seldom been tested. We aimed to elucidate the association between uric acid and cerebral microbleeds (CMBs), a hemorrhage-prone cerebral microangiopathy. Seven hundred twenty-four patients with ischemic stroke who were consecutively admitted to our hospital were included in this study. We collected demographic, clinical, and laboratory data, including uric acid level, and examined the presence of CMBs using T2*-weighted gradient-echo MRI. We used logistic regression analysis to examine an independent association between uric acid and CMBs. Two-hundred twenty-six patients had CMBs (31.2%). After adjusting for possible confounders, elevated uric acid was independently associated with the presence of CMBs (the highest quartile vs. lowest quartile, adjusted odd ratio [OR], 1.98; 95% confidence interval [CI], 1.16–3.39). This association retained in patients with deep or infratentorial CMBs (with or without lobar CMBs) but not among those with lobar CMBs. In addition, this association was robust among patients with hypertension (the highest quartile vs. lowest quartile, adjusted OR, 2.74; 95% CI, 1.43–5.24). In contrast, we did not find the association in patients without hypertension. We demonstrated that serum uric acid is independently associated with the presence of CMBs. In particular, the relation between uric acid and CMBs was robust in hypertensive patients.

## Introduction

Uric acid has been reported to exert neuroprotective effects by acting as a free radical scavenger [1]. Uric acid is a strong reducing agent and a potent antioxidant, and approximately one half of the antioxidant capacity of plasma comes from uric acid [2]. In an animal stroke model, the administration of uric acid resulted in a better outcome [3]. In contrast, large population-based studies have indicated that increased levels of uric acid are an independent risk factor for cardiovascular disease and stroke [4], [5]. Elevated uric acid levels have been associated with a poor outcome in patients with heart failure [6], coronary heart disease [7], and stroke [8]. The precise role of uric acid in vascular disease is still a matter of ongoing controversy.

Subclinical vascular brain lesions are easily visualized by brain magnetic resonance image (MRI). The lesion findings are generally classified into ischemia-prone and hemorrhage-prone microangiopathy [9]. The former has been referred as white matter lesions (WMLs) or leukoaraiosis seen on T2-weighted or fluid-attenuated inversion recovery MRI [10]. The latter was recently identified, and has been frequently called cerebral microbleeds (CMBs). Because the nature of these lesions is small bleeding from the advanced lipohyalinized arterioles due to chronic hypertension, the CMBs have been understood to be a harbinger of intracerebral hemorrhage (ICH) [11]–[13].

A recent report has indicated that increased levels of uric acid are positively associated with large WMLs [4], but there has been no study on the relationship between uric acid and CMBs. Given a proven association between uric acid and vascular disease, we hypothesized that levels of uric acid are related with the presence of CMBs. In this study, we sought to find an association between levels of uric acid and presence of CMBs in a large-sized consecutive series of ischemic stroke patients.

## Methods

### Study Population

Between March 2003 and September 2006, we consecutively enrolled acute ischemic stroke patients aged 50 years or older who were admitted to our hospital within 7 days after symptom onset (n = 834). We excluded patients who did not undergo gradient-echo MR imaging for various reasons (n = 95) or lacked essential medical information (n = 15). The final sample size was 724 stroke patients recruited for this study. The study was carried out in accordance with the Declaration of Helsinki and was approved by the Seoul National University Hospital institutional board review with waiver of consent.

### Blood Pressure Measurement and Data Collection

We recorded demographic data, conventional risk factors, and important laboratory data for all subjects. Conventional risk factors included hypertension, diabetes, hypercholesterolemia, heart diseases, and smoking history. Blood pressure was measured on the discharge day. After a 5-minute period of supine rest, blood pressures were measured by auscultation in the dominant arm using a random-zero sphygmomanometer on 2 occasions separated by 2 minutes and averaged. If the dominant arm was affected by stroke, we used the non-dominant arm for a blood pressure measurement. Hypertension was diagnosed as present if the patients exhibited a systolic blood pressure >140 mm Hg or a diastolic blood pressure >90 mm Hg, or had a history of diagnosis of hypertension and anti-hypertensive medications. Only 4.1% patients had a blood pressure measurement within 7 days after symptom onset. Diabetes was present if subjects exhibited a fasting glucose level >7.0 mmol/L (126 mg/dL) or had a history of diabetes diagnosis and anti-diabetic medications. A diagnosis of hypercholesterolemia was made for patients with a history of using cholesterol-lowering agents or who had a fasting serum total cholesterol level >6.2 mmol/L (240 mg/dL) on admission. Heart diseases included atrial fibrillation, myocardial infarction, and valvular heart disease. Smoking was coded as positive if the patient was a current smoker or an ex-smoker who had quit smoking within five years prior to stroke onset. Fasting blood samples were drawn within 24 h of admission, and examined for uric acid and a standard battery of biochemical and hematological tests. Uric acid level was measured by uric acid oxidase reagent on a Dax analyzer (Bayer-Technichon) [14]. Glomerular filtration rate was calculated using a 4-item Modification of Diet in Renal Disease formula [15].

### Brain MR Imaging

Brain MR imaging was performed on a 1.5 T superconducting magnet system (GE Medical System, Milwaukee, WI, USA). T2*-weighted gradient-echo MR imaging data were obtained in the axial plane using the following parameters: repetition time/echo time, 500/15 msec; flip angle, 26°; and matrix size, 256×192. CMBs were characterized as well-defined focal areas of dark signal intensity measuring less than 5 mm in diameter with blooming artifact on gradient-echo MR imaging. We did not include lesions that were within the subarachnoid space and areas of symmetric hypointensity of the globus pallidus, which were likely to represent adjacent pial blood vessels and calcification, respectively. CMBs were categorized in one of three locations: lobar (cortical gray and subcortical or periventricular white matter), deep (deep gray matter: basal ganglia and thalamus, and the white matter of the corpus callosum, internal, external, and extreme capsule), and infratentorial (brainstem and cerebellum) [16]. Two stroke neurologists (B.J. Kim and C.K. Kim), blinded to the clinical characteristics, reviewed the images.

Fluid-attenuated inversion recovery MR imaging was performed to detect WMLs using the following parameters: repetition time/echo time, 8500/96 ms; inversion time, 2100 ms; flip angle, 26°; and matrix size, 256×192. The WMLs were classified into four grades: grade 0, no abnormality or minimal periventricular signal hyperintensities in the form of caps confined exclusively to the anterior horns or rims lining the ventricles; grade 1, hyperintensities in both the anterior and posterior horns of the lateral ventricles or periventricular unifocal patches; grade 2, multiple periventricular hyperintense punctate lesions and their early confluence; and grade 3, multiple areas of high signal intensity reaching confluence in the periventricular region Interrator reliabilities for CMBs and WMLs were 0.81 and 0.74, respectively [17].

### Statistical Analysis

As serum uric acid levels substantially differed between men and women, we divided the patients into sex-specific quartile with cut-points of 0.24, 0.30, and 0.37 mmol/L for men. The corresponding values for women were 0.20, 0.26, and 0.32 mmol/L. The differences in clinical and demographic characteristics were compared using *t*-tests, χ^2^ tests, or one-way analysis of variance as appropriate. Multivariable logistic regression models were used to determine the independent association between uric acid quartiles and CMBs. We selected covariates that are related with the presence of CMBs in univariate analysis with p<0.10. In addition, we also incorporated predefined potential confounders that are acknowledged to be associated with CMBs in the prior studies. The covariates included age, sex, hypertension, diabetes, previous stroke, heart disease, previous antithrombotic use, glomerular filtration rate, body mass index, smoking, and total cholesterol. We further included WMLs as a covariate to examine an independent association between levels of uric acid and CMBs, because CMBs and WMLs are closely related pathologically, and may have a statistical interaction. To assess the different impacts of uric acid on CMBs by location, we repeated the analyses in separate groups: strictly lobar CMBs and deep or infratentorial CMBs (with or without lobar CMBs). Considering the relation between uric acid and hypertension, we also examined whether the association between CMBs and uric acid is dissimilar by the presence of hypertension. For the sensitivity analysis, we reran the multivariable model after excluding the patients with blood pressure measurement <7 days after symptom onset. A two-tailed *p* value of <0.05 was considered to be statistically significant. Data analyses were performed using SPSS ver.12.0 (SPSS Inc., Chicago, IL, USA).

## Results

The uric acid level ranged from 0.06 to 0.71 mmol/L (mean, 0.30±0.10 mmol/L). Baseline characteristics according to the presence of CMBs are elaborated in Table 1. Patients with CMBs were more likely to be older and to have hypertension, a history of previous stroke, higher WMLs grade, and higher systolic blood pressure compared with those without CMBs. Patients with higher quartiles of uric acid were likely to have hypertension, hypercholesterolemia, and heart disease (Table 2). The grade of WMLs tended to increase with increasing quartiles, albeit not significant.

**Table 1:** Baseline Characteristics of Patients According to Presence or Absence of CMBs.

Variables	All (n = 724)		
	Without CMBs (n = 498)	With CMBs (n = 226)	*p*
Age	66.9±8.9	68.5±8.9	0.02
Hypertension	63.9%	80.1%	<0.01
Diabetes	35.9%	27.8%	0.03
Hypercholesterolemia	18.0%	14.9%	0.30
Current smoking	28.7%	22.4%	0.07
Previous stroke	19.0%	27.0%	0.01
Heart disease	29.0%	28.6%	0.90
Previous use of antithrombotic	13.6%	13.8%	0.94
SBP (mmHg)	139±23	145±25	<0.01
DBP (mmHg)	83±14	87±17	<0.01
BMI (kg/m^2^)	24.0±3.1	24.1±3.1	0.92
GFR (mL/min/1.73 m^2^)	68.7±21.0	67.3±19.5	0.41
White matter lesions grade*			
0	37.9%	14.5%	<0.01
1	37.5%	30.8%	
2	16.5%	35.0%	
3	8.1%	19.7%	
Uric acid (mmol/L)	0.29±0.99	0.32±0.11	<0.01
Glucose (mmol/L)	6.65±2.33	6.60±2.78	0.79
Cholesterol (mmol/L)	4.73±0.99	4.74±1.06	0.84

Data are presented as means±SD or percentages.

CMBs, cerebral microbleeds; BMI, body mass index; SBP, systolic blood pressure; DBP, diastolic blood pressure; GFR, glomerular filtration rate.

* White matter lesions were graded as absent (0), punctuate (1), early confluent (2), or confluent (3).

**Table 2:** Baseline Characteristics of Patients across Sex-Specific Quartile of Uric Acid.

	Uric Acid (N = 724)				
	Quartile 1 (n = 186)	Quartile 2 (n = 183)	Quartile 3 (n = 170)	Quartile 4 (n = 185)	*p*
Presence of CMBs	23.1%	25.7%	35.9%	40.5%	<0.01
Age	67.8±9.0	66.4±9.3	67.7±8.9	67.7±9.0	0.44
Sex, men	60.8%	65.0%	64.1%	67.0%	0.65
Hypertension	56.5%	60.7%	75.9%	80.5%	<0.01
Diabetes	30.6%	38.2%	37.2%	30.8%	0.27
Hypercholesterolemia	11.8%	14.8%	17.6%	22.7%	0.04
Previous stroke	23.7%	17.5%	21.8%	22.7%	0.49
Heart disease	22.6%	31.7%	20.0%	40.0%	<0.01
Smoking (current or quit <5 years)	23.1%	34.4%	26.5%	26.5%	0.10
BMI (kg/m^2^)	23.2±3.0	24.2±3.6	24.3±3.0	24.2±3.0	<0.01
Previous use of antithrombotic	13.1%	15.4%	11.4%	13.2%	0.75
Systolic blood pressure, mmHg	140±25	139±22	144±25	141±24	0.19
Diastolic blood pressure, mmHg	83±14	84±14	85±17	86±15	0.17
Total cholesterol, mmol/L	4.63±0.96	4.62±0.88	4.83±0.94	4.79±1.16	0.09
Glucose, mmol/L	6.83±2.66	7.05±2.78	6.48±2.29	6.43±2.18	0.07
GFR (mL/min/1.73 m^2^)	66.4±18.7	68.7±23.9	69.8±18.9	68.1±19.8	0.50
White matter lesion grade					0.18
0	35.1%	39.0%	24.1%	27.2%	
1	31.9%	31.9%	36.1%	35.9%	
2	22.7%	18.7%	25.9%	23.9%	
3	10.3%	10.4%	13.9%	13.0%	

Data are presented as means±SD or percentages.

CMBs, cerebral microbleeds; BMI, body-mass index.

The prevalence of CMBs increased with increasing quartiles (Table 3). After adjustment for age and sex, the third and the fourth quartiles of uric acid were significantly associated with the presence of CMBs. After adjusting for other potential confounders, the adjusted odds ratio of CMBs for the third and the fourth quartiles were 1.78 (95% confidence interval [CI], 1.06–2.98) and 1.93 (95% CI, 1.15–3.32). Although adjustment for WMLs moderately attenuated the association, the patients in the highest quartile of uric acid had a 2-fold increased risk of CMBs compared with those in the lowest quartile. In addition, age (per one year, odd ratio [OR] 1.04, 95% CI 1.01–1.07), previous history of stroke (OR 2.00, 95% CI 1.16–3.45), and hypertension (OR 2.28, 95% CI 1.43–3.65) were independently associated with the presence of CMBs.

**Table 3:** Multivariable Logistic Regression Analysis for Presence of Cerebral Microbleeds.

	Patients withCMBs, %	Age and sex adjustedOR (95% CI)	Model 1 adjustedOR (95% CI)	Model 2 adjustedOR (95% CI)
Any (n = 226)				
Quartile 1	23.1%	Reference	Reference	Reference
Quartile 2	25.7%	1.19 (0.74–1.92)	1.05 (0.60–1.82)	1.07 (0.60–1.91)
Quartile 3	35.9%	1.89 (1.19–3.01)	1.78 (1.06–2.98)	1.65 (0.96–2.83)
Quartile 4	40.5%	2.33 (1.48–3.66)	1.93 (1.15–3.23)	1.98 (1.16–3.39)
P for trend		0.001	0.017	0.033
Strictly Lobar (n = 46)				
Quartile 1	5.9%	Reference	Reference	Reference
Quartile 2	8.2%	1.43 (0.64–3.21)	1.42 (0.59–3.42)	1.44 (0.60–3.50)
Quartile 3	5.3%	0.89 (0.36–2.20)	1.13 (0.44–2.93)	1.16 (0.45–3.00)
Quartile 4	5.9%	1.00 (0.42–2.38)	1.00 (0.39–2.58)	1.01 (0.39–2.61)
*P* for trend		0.676	0.845	0.826
Deep or infratentorial* (n = 180)				
Quartile 1	17.2%	Reference	Reference	Reference
Quartile 2	17.5%	1.06 (0.62–1.82)	0.83 (0.43–1.60)	0.81 (0.40–1.62)
Quartile 3	30.6%	2.16 (1.31–3.58)	1.85 (1.05–3.27)	1.67 (0.92–3.03)
Quartile 4	34.6%	2.62 (1.61–4.28)	2.14 (1.22–3.77)	2.21 (1.22–4.00)
*P* for trend		<0.001	0.003	0.005

Model 1 is adjusted for age, sex, hypertension, diabetes, previous stroke, heart disease, previous antithrombotic use, glomerular filtration rate, body mass index, smoking and total cholesterol; model 2, adjusted for model 1 plus white matter lesion.

* with or without lobar cerebral microbleeds.

When we divided CMBs into strictly lobar and deep or infratentorial (with or without lobar CMBs), the association between uric acid quartiles and CMBs was significant in the deep or infratentorial CMBs group but not in the strictly lobar CMBs group (Table 3). In fully adjusted model, ORs of deep of infratentorial CMBs for the fourth quartile of uric acid was 2.21 (95% CI, 1.22–4.00). We found a significant relationship of uric acid with CMBs in patients with hypertension but not among those without hypertension. In patients with hypertension, the third and the fourth quartiles of uric acid were independently associated with CMBs in fully adjusted model (adjusted ORs 1.92 and 2.74, table 4).

**Table 4:** Multivariable Logistic Regression Analysis for Presence of Cerebral Microbleeds in Patients with or without Hypertension.

	Patients withCMBs, %	Age and sex adjustedOR (95% CI)	Model 1 adjustedOR (95% CI)	Model 2 adjustedOR (95% CI)
Without hypertension (n = 226)				
Quartile 1	17.3%	Reference	Reference	Reference
Quartile 2	19.4%	1.24 (0.54–2.86)	0.88 (0.30–2.57)	0.78 (0.26–2.39)
Quartile 3	19.5%	1.19 (0.45–3.14)	1.33 (0.45–3.99)	1.22 (0.39–2.78)
Quartile 4	19.4%	1.17 (0.42–3.23)	0.82 (0.24–2.80)	0.73 (0.20–2.65)
P for trend		0.963	0.882	0.863
With hypertension (n = 494)				
Quartile 1	27.6%	Reference	Reference	Reference
Quartile 2	29.7%	1.11 (0.62–2.02)	0.99 (0.49–2.02)	1.06 (0.50–2.21)
Quartile 3	41.1%	1.87 (1.07–3.26)	2.04 (1.08–3.88)	1.92 (1.05–3.70)
Quartile 4	45.6%	2.27 (1.32–3.88)	2.59 (1.39–4.82)	2.74 (1.43–5.24)
P for trend		0.005	0.002	0.004

Model 1 is adjusted for age, sex, diabetes, previous stroke, heart disease, previous antithrombotic use, body mass index, glomerular filtration rate, smoking, and total cholesterol; model 2, adjusted for model 1 plus white matter lesion.

Thirty patients had a blood pressure measurement within 7 days after symptom onset because of in-hospital death (n = 21) and early discharge due to mild symptom (n = 9). After exclusion of these patients, the association between CMBs and uric acid essentially unchanged. In fully adjusted model, the odds ratio of CMBs for the third and the fourth quartiles were 1.77 (95% confidence interval [CI], 1.03–2.96) and 1.94 (95% CI, 1.18–3.41).

## Discussion

We found that uric acid is independently associated with the presence of CMBs in patients with acute ischemic stroke. The association sustained in patients with deep or infratentorial CMBs but not among those with strictly lobar CMBs. Furthermore, this association was strengthened in patients with hypertension. In patients without hypertension, however, uric acid was not related to the presence of CMBs.

CMBs are divided into two subtypes according to its main pathology, hypertensive microangiopathy and cerebral amyloid angiopathy [9]. CMBs related with hypertensive predominantly located at deep brain structure, basal ganglia, thalamus, and brain stem. In contrast, CMBs related with cerebral amyloid angiopathy are commonly found in subcortical area. For hypertensive CMBs, chronic hypertension is the most consistent and important risk factor [16], [18]. Moreover, hypertensive CMBs have been associated with hypertensive organ damage, including left ventricular mass index [19], hypertensive retinopathy [20], and chronic kidney disease [21]. Several pathophysiological mechanisms linking uric acid to hypertensive organ damage at the cellular and tissue levels have been proposed, including a proliferation of vascular smooth muscle cells [22], stimulation of the inflammatory pathway [23], and possible prothrombotic effects mediated by platelet activation [24]. Furthermore, uric acid has proven to be an excellent marker for tissue ischemia and endothelial dysfunction [25], [26], and it has been shown to play a role in the evolution of atherosclerotic lesions [27]. Analyses based on the Framingham Heart Study have shown that serum uric acid was an independent predictor of the presence and progression of hypertension [28]. Taken together, our results that uric acid is associated with deep or infratentorial CMBs but not with lobar CMBs and that the relationship between uric acid and CMBs was found in patients with hypertension but not in those without hypertension suggest that CMBs may be the brain phenotype of hypertensive organ damage and uric acid may in part mediate the injury process.

Ample evidence has indicated that CMBs are closely related to ICH [11], [29]. Earlier studies have suggested that CMBs could be a representative marker of ICH based on reports that the risk factors for CMBs are similar to those for ICH and on the common histopathologic findings for both types of lesions [30], [31]. Furthermore, recent reports have strongly corroborated the association between CMBs and ICH: (1) Regional association between CBMs and ICH [13]; (2) Association between CMBs and volume, occurrence, and recurrence of ICH [32], [33]; (3) Relationship of CMBs with warfarin-related hemorrhage [11]; and (4) Association between CMBs and the hemorrhagic transformation after thrombolysis [34]. Hence, it seems plausible to assume that increased levels of uric acid might increase a risk of ICH.

In the present study, we found no significant association between uric acid and WMLs. This is supposedly caused by a crude white matter lesion grading system. In a previous study demonstrating a positive association between uric acid and WMLs, the WMLs were calculated in a quantitative manner [4]. However, we graded WMLs according to the system described by Fazekas [35].

Several limitations of our study deserve comment. First, it is well known that patients with acute stroke, even without a history of previous hypertension, often have a high blood pressure [36]. Previous studies have shown that blood pressure gradually declines and reaches baseline level at 7 days after symptom onset [36], [37]. Although only 4.1% patients in our study had a blood pressure measurement within 7 days after symptom onset and the association between uric acid and CMBs sustained after excluding these patients, we could not completely rule out the possibility that a blood pressure variation caused by acute stroke has influenced out results. Second, our study included patients with acute ischemic stroke. The presence of CMBs could therefore be affected by acute ischemic stroke [38]. However, a previous report have revealed that a rapid appearance of CMBs after ischemic stroke was related with only the presence of baseline CMBs and white matter lesion volumes. Thus, we believe that this bias had little impact on our results [38]. Third, there is a possibility of selection bias. To minimize the likelihood of bias, we studied all consecutive patients, and uric acid was measured as a part of the routine work-up. Fourth, we were unable to obtain the information as to whether the patients had used uric acid-lowering agents. After a meticulous search in the medical records, we found some patients who were taking uric acid-lowering agents. However, this investigation raised a possibility of an additional bias because we were unable to inspect the entire sample due to the retrospective nature of our study. Finally, our hypothesis-generating cross-sectional study was unable to provide a direct causal relationship between uric acid and CMBs.

Although it is indeterminate whether hyperuricemia is an independent risk factor requiring treatment, or if it is an innocent bystander in proximity to vascular accidents that merely reflects an adverse risk factor pattern, recent evidence suggests that uric acid may be deleterious to the brain rather than protective [5], [8], [39]–[42]. Given that the presence of CMBs portends an increased risk of ICH our study suggests another detrimental role of uric acid as a potential ICH risk factor.
